# Rapid reaction studies on the chemistry of flavin oxidation in urocanate reductase

**DOI:** 10.1016/j.jbc.2024.105689

**Published:** 2024-01-26

**Authors:** Niusha Delavari, Zhiyao Zhang, Frederick Stull

**Affiliations:** Department of Chemistry, Western Michigan University, Kalamazoo, Michigan, USA

**Keywords:** flavoenzyme, flavoprotein, flavin, urocanate reductase, reductase, enzyme kinetics, stopped-flow, oxidative half-reaction, mutagenesis, isotope effects, catalytic mechanism, urocanate, imidazole propionate, diabetes

## Abstract

Urocanate reductase (UrdA) is a bacterial flavin-dependent enzyme that reduces urocanate to imidazole propionate, enabling bacteria to use urocanate as an alternative respiratory electron acceptor. Elevated serum levels of imidazole propionate are associated with the development of type 2 diabetes, and, since UrdA is only present in humans in gut bacteria, this enzyme has emerged as a significant factor linking the health of the gut microbiome and insulin resistance. Here, we investigated the chemistry of flavin oxidation by urocanate in the isolated FAD domain of UrdA (UrdA′) using anaerobic stopped-flow experiments. This analysis unveiled the presence of a charge-transfer complex between reduced FAD and urocanate that forms within the dead time of the stopped-flow instrument (∼1 ms), with flavin oxidation subsequently occurring with a rate constant of ∼60 s^−1^. The pH dependence of the reaction and analysis of an Arg411Ala mutant of UrdA′ are consistent with Arg411 playing a crucial role in catalysis by serving as the active site acid that protonates urocanate during hydride transfer from reduced FAD. Mutational analysis of urocanate-binding residues suggests that the twisted conformation of urocanate imposed by the active site of UrdA′ facilitates urocanate reduction. Overall, this study provides valuable insight into the mechanism of urocanate reduction by UrdA.

Elevated serum levels of the histidine metabolite, imidazole propionate (ImP), have recently been associated with prediabetes and the development of type 2 diabetes in humans (1, 2, 3). This metabolite appears to activate mechanistic target of rapamycin complex 1, thereby impairing insulin signaling and glucose tolerance (1). ImP is produced by the enzyme urocanate reductase (UrdA), which is only found in bacteria, including those in the gut microbiome (1, 4, 5). An altered gut microbiome is known to be associated with type 2 diabetes (6, 7), including enrichment in bacteria with the UrdA gene, which presumably leads to the elevated ImP levels in individuals with type 2 diabetes, thus providing a link between microbial metabolism, UrdA activity, and the metabolic disorder insulin resistance (5, 6, 7, 8). Urocanate is produced from histidine catabolism by histidine ammonia lyase, and UrdA allows bacteria to use urocanate as the terminal electron acceptor of a respiratory electron transport chain. In this pathway, UrdA catalyzes the unidirectional reduction of urocanate to ImP, enabling anaerobic growth of bacteria containing UrdA when urocanate is present in the environment (4, 9). Since ImP is only produced in humans by bacterial UrdA in the gut, UrdA may be a potential therapeutic target for reducing the severity of type 2 diabetes.

Full-length UrdA possesses two domains and two prosthetic groups: FMN is covalently attached to a serine residue in the N-terminal domain and FAD is noncovalently bound to the C-terminal domain (4). The N-terminal domain is also predicted to be covalently attached to the bacterial membrane, and electrons are thought to flow from electron donors in the membrane to FMN, then to FAD in the C-terminal domain where urocanate reduction to ImP occurs during anaerobic respiration on urocanate (Fig. 1) (9). The isolated C-terminal domain (hereafter referred to as UrdA′) is catalytically active toward urocanate reduction when supplied with an artificial electron donor, and structures have been solved for UrdA′ from *Shewanella oneidensis* MR-1 in the absence of ligands and with urocanate and ImP bound in the active site (10). The structure of UrdA′ is similar to the well-characterized soluble flavocytochrome c_3_ fumarate reductase (Fcc_3_) from *Shewanella* species (Figs. 2 and S1), which allows bacteria to respire using fumarate as the final electron acceptor (11, 12, 13). In both enzymes, the substrate is bound above the isoalloxazine of FAD, in a favorable position for C3 of the substrate to receive a hydride from N5 of the isoalloxazine in reduced FAD (FADH_2_). Arg401/402 in *Shewanella* sp. Fcc3 is the active site acid that protonates C2 of the substrate alongside hydride transfer, and evidence suggests that this arginine gets reprotonated during catalysis by a proton transfer pathway involving Glu377/378 and Arg380/381 (12). UrdA′ also has an arginine residue (Arg411) at the same position as Arg401/402 Fcc3, suggesting that Arg411 in UrdA′ may play a similar role as an active site acid to complete urocanate reduction by UrdA. Curiously, UrdA′ has Ala387 in place of Glu377/378 in Fcc3, and no other residue is appropriately positioned to participate in a similar proton transfer pathway as that found in Fcc3 (Fig. 2). However, Arg411 is more solvent accessible in the structure of UrdA′ than in Fcc3, suggesting that a proton transfer pathway may not be necessary for catalysis in UrdA (10).

**Figure 1:**
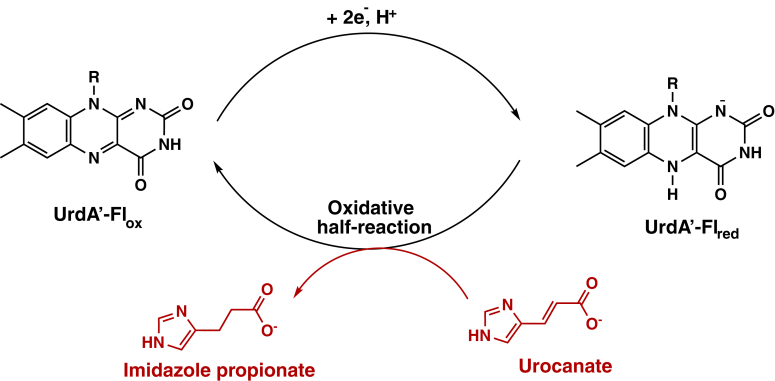
**Summarized half-reactions of urocanate reductase (UrdA)**.

**Figure 2:**
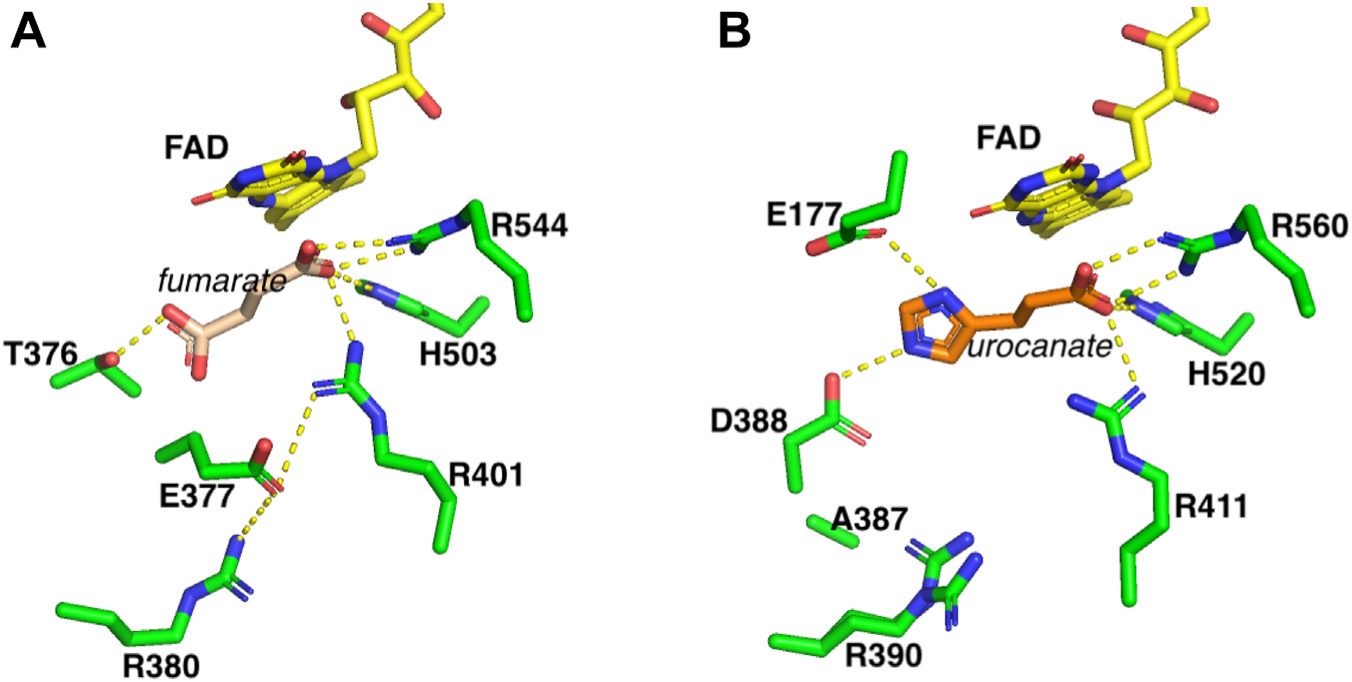
**Comparison of active sites.** Active site of (*A*) fumarate reductase in complex with fumarate (Protein Data Bank ID: 1D4E) and (*B*) urocanate reductase in complex with urocanate (Protein Data Bank ID: 6T87).

While structures have illuminated important aspects of the UrdA active site, mechanistic understanding of UrdA’s catalytic activity is lacking. Here, we have investigated the mechanism of urocanate reduction by UrdA′ using anaerobic stopped-flow methods. In rapid reaction studies of the reaction of reduced UrdA′ with urocanate, we have detected the presence of a reduced flavin–urocanate charge-transfer complex prior to flavin oxidation. Our kinetic data also support the role of Arg411 as the proton donor in facilitating urocanate reduction by UrdA′, and that the twisted conformation of urocanate in the active site may be important for catalysis.

## Results

Our study used a UrdA variant (UrdA′) that lacks the membrane-anchored N-terminal domain (FMN domain). Unlike the full-length UrdA, UrdA′ can be expressed and purified from *Escherichia coli* without requiring covalent flavinylation (10). However, it retains the FAD containing urocanate-binding domain, which has been shown to be catalytically active, and for which crystal structures have been solved (10).

**Figure 3:**
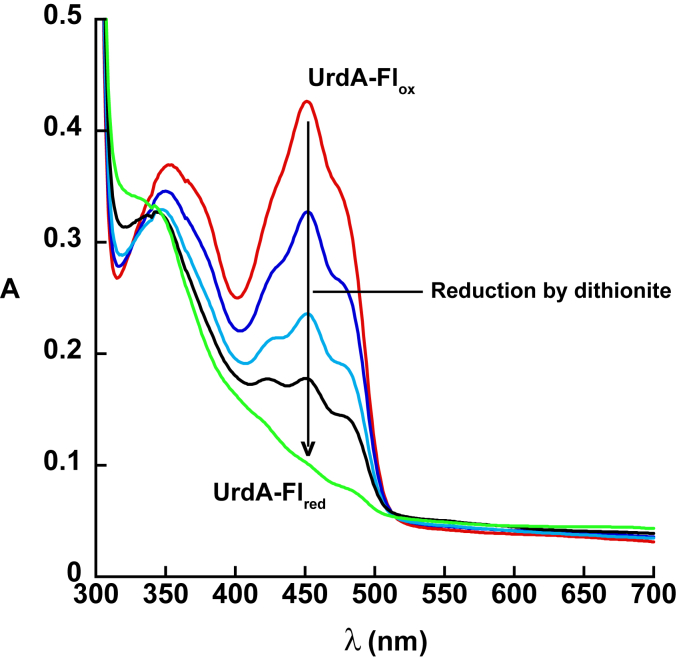
**Dithionite reduction of UrdA’.** UV–visible absorbance changes observed in the region of FAD absorbance after anaerobic reduction of 35 μM UrdA′-Fl_ox_ by sodium dithionite. The λ_max_ is 452 nm for oxidized enzyme. UrdA, urocanate reductase.

Using spectrophotometry, it is possible to monitor the oxidation state of the flavin cofactor of UrdA′ (14). UrdA′ shows a typical absorbance spectrum for oxidized FAD (UrdA′-Fl_ox_) when purified. The physiological reductant for UrdA is currently unknown. However, the oxidized flavin of UrdA′-Fl_ox_ can be reduced to the flavin hydroquinone (UrdA′-Fl_red_) by anaerobic titration with dithionite. During the titration with dithionite, UrdA′-Fl_ox_ reduced directly to UrdA′-Fl_red_ without forming a flavin semiquinone intermediate (Fig. 3). UrdA′-Fl_red_ prepared by anaerobic reduction with dithionite was used in all experiments on the oxidative half-reaction for UrdA′ in this study.

### Oxidative half-reaction

**Figure 4:**
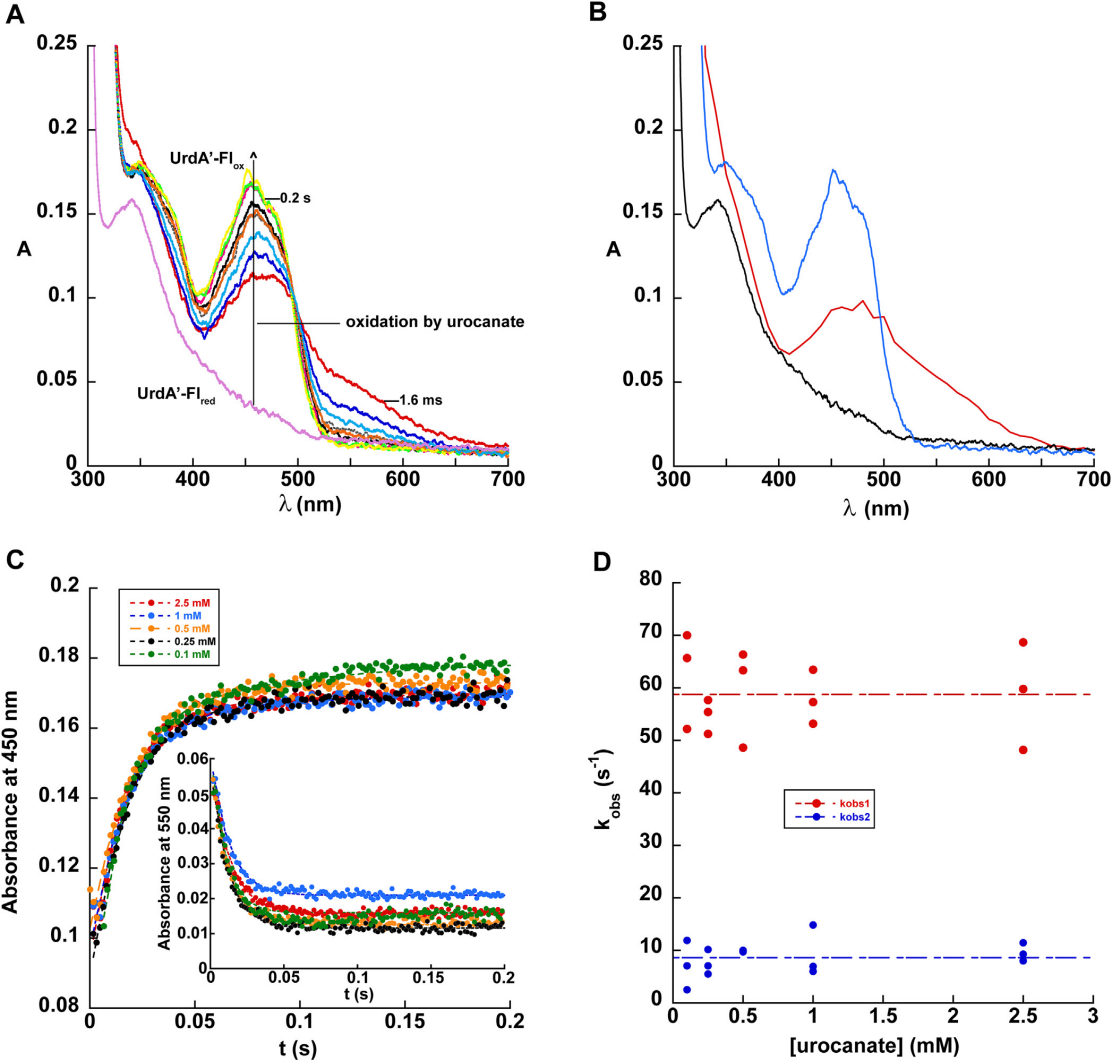
**Reaction between UrdA′-Fl**_**red**_**and urocanate.***A*, 15 μM UrdA′-Fl_ox_ was anaerobically reduced with dithionite, mixed with different concentrations of urocanate, and monitored for changes in FAD absorbance at pH 7, 4 °C using stopped-flow spectrophotometry. Each spectrum shows a different time point spanning from 1.6 ms to 0.2 s. The spectrum of UrdA′-Fl_red_ prior to mixing with urocanate is also shown for comparison. *B*, comparison of the absorbance spectra between UrdA′-Fl_ox_ (*blue*), UrdA′-Fl_red_ (*black*), and the intermediate formed within the dead time in the reaction between UrdA′-Fl_red_ and urocanate (*red*). *C*, absorbance trace overlay at 450 nm for the oxidation of wildtype UrdA′-Fl_red_ by different concentrations of urocanate at pH 7. The *inset* shows an absorbance trace overlay at 550 nm. *D*, *k*_obs_ values for the first and second phases of the reaction plotted against the urocanate concentration. UrdA, urocanate reductase.

**Figure 5:**
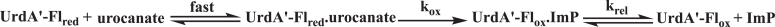
**Proposed kinetic model for the reaction between UrdA′-Fl**_**red**_**and urocanate.** UrdA, urocanate reductase.

We employed a stopped-flow instrument to measure the kinetics of the oxidative half-reaction of UrdA′ at pH 7 (Fig. 1). UrdA′-Fl_red_ was mixed with excess urocanate in the absence of O_2,_ and the progress of the reaction was tracked by following the flavin absorbance over time using the instrument's multiwavelength charge-coupled device detector. The first absorbance scan at 1.6 ms obtained immediately after mixing UrdA′-Fl_red,_ and urocanate exhibited distinct differences compared with the absorbance spectrum of UrdA′-Fl_red_ alone (Fig. 4*A*), indicating that an intermediate was formed within the dead time of the instrument. Extrapolating back to time 0 at all wavelengths showed that this intermediate has an absorbance spectrum that resembles other charge-transfer complexes involving reduced flavin and an electron-deficient ligand, with a broad absorbance that extends out to 650 nm (Fig. 4*B*) (15, 16). The most straightforward explanation for this absorbance change and the resulting charge-transfer signal is that urocanate binding to UrdA′-Fl_red_ occurred in the dead time of the instrument (Fig. 5). After this initial charge-transfer complex, we observed the generation of oxidized flavin and the simultaneous disappearance of the charge-transfer band at long wavelengths.

**Figure 6:**
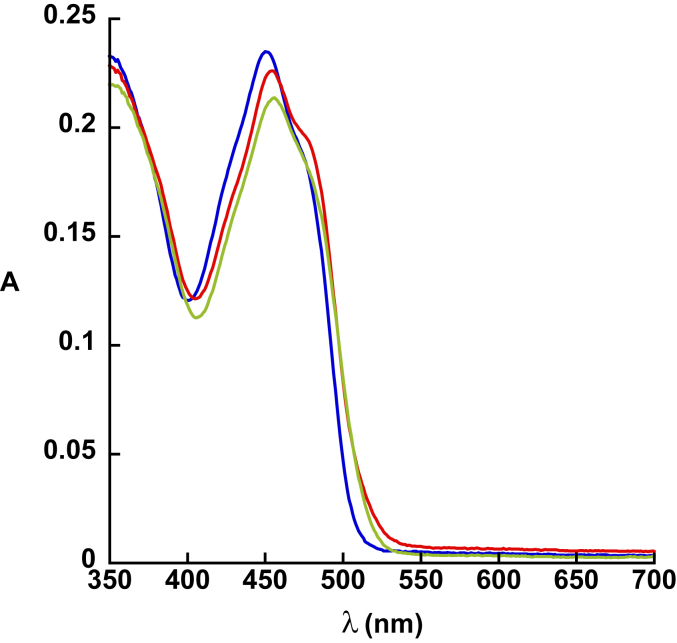
**Absorbance spectra of UrdA′-Fl**_**ox**_**complexes at pH 7.** Absorbance spectra for UrdA′-Fl_ox_ alone (*blue*), the UrdA′-Fl_ox_–urocanate complex (*red*, λ_max_ = 454 nm), and the UrdA′-Fl_ox_–imidazole propionate complex (*green*, λ_max_ = 455 nm). The UrdA′ concentration was 20 μM in all samples, and 200 μM ligand was added for the spectra of each complex. UrdA, urocanate reductase.

The reaction was completed in less than 1 s and based on the reaction traces observed at 450 nm (Fig. 4*C*), the most suitable fit was given by a double exponential function (Equation 2). The first exponential contributed ∼95% of the signal change at 450 nm and therefore corresponds to the oxidation of UrdA′-Fl_red_ to UrdA′-Fl_ox_ by urocanate, which gets converted into ImP. Traces at 550 nm show a decrease in absorbance that fits to a single exponential function (Equation 1) with a comparable observed rate constant (*k*_obs_) to the first exponential at 450 nm (Fig. 4*C*, *inset*). We attribute the second phase at 450 nm to the rate-limiting release of the ImP product from UrdA′-Fl_ox_ followed by rapid association of urocanate, as the absorbance at 450 nm of the UrdA′-Fl_ox_–urocanate complex is about 5% higher than that of the UrdA′-Fl_ox_–ImP complex (Fig. 6). The observed rate constant for conversion of UrdA′-Fl_red_ to UrdA′-Fl_ox_ by urocanate was invariant at 59 s^−1^ across all urocanate concentrations tested (Fig. 4*D*), indicating that the dissociation constant (*K*_*d*_) for urocanate binding to UrdA′-Fl_red_ is considerably lower than the lowest urocanate concentration used in our experiment (100 μM), consistent with the high affinity reported previously for full-length UrdA (4). Note that we cannot sample lower urocanate concentrations and still maintain the pseudo-first order conditions necessary for analysis. Similarly, the *k*_obs_ for release of the ImP product from UrdA′-Fl_ox_ (*k*_rel_) was invariant at 8.6 s^−1^ at all urocanate concentrations used (Fig. 4*D*).

**Figure 7:**
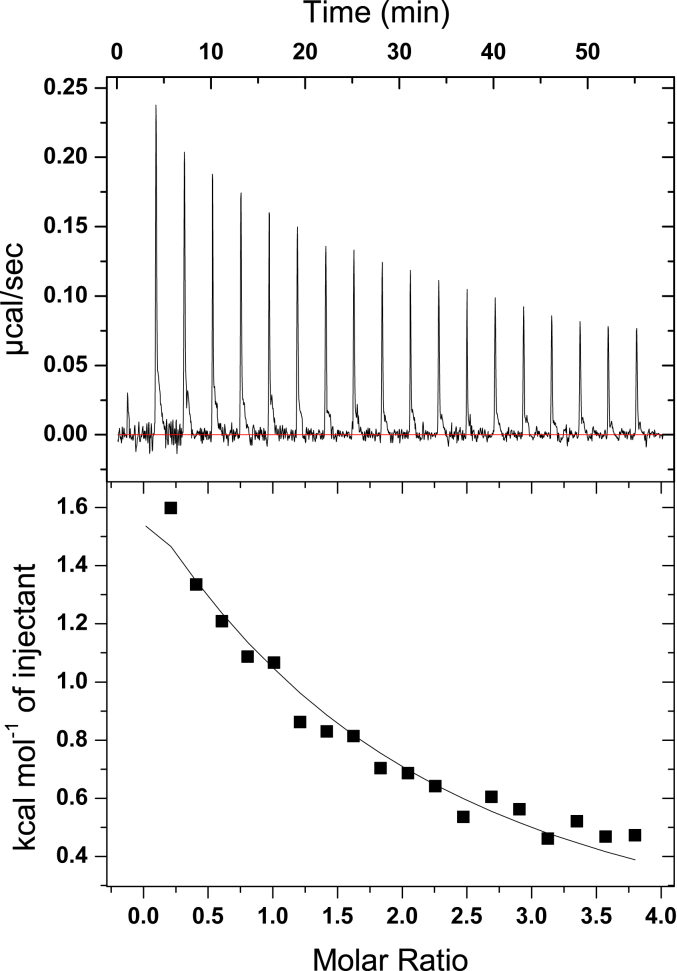
**Isothermal titration calorimetry (ITC) measurement of urocanate binding to UrdA′ at 4**°C. *Upper panel*, baseline-corrected thermogram for the titration. *Lower panel*, the *black squares* show the integrated heats for each injection, and the *black line* shows the fit to a one-site model. The fit provided a *K*_*d*_ of 160 ± 20 μM and a binding enthalpy (ΔH) of 6.4 ± 0.4 kcal/mol. UrdA, urocanate reductase.

The low *K*_*d*_ value for urocanate indicated by our stopped-flow data is at odds with the 280 μM *K*_*d*_ value reported for urocanate binding by isothermal titration calorimetry (ITC) in another study (10). Our experimental conditions are distinct from those used in the prior study, and we therefore measured the *K*_*d*_ value for urocanate binding to UrdA′-Fl_ox_ by ITC using our experimental conditions. The binding affinity under our experimental conditions for urocanate binding to UrdA′-Fl_ox_ was 160 μM (Fig. 7). The considerably smaller *K*_*d*_ value indicated by our kinetic analysis of the reaction between UrdA′-Fl_red_ and urocanate suggests that UrdA binds urocanate with a higher affinity when its FAD is in the reduced hydroquinone state than it does when the flavin is oxidized.

### pH dependence and determination of the microscopic p*K*_a_ of the oxidative half-reaction

**Figure 8:**
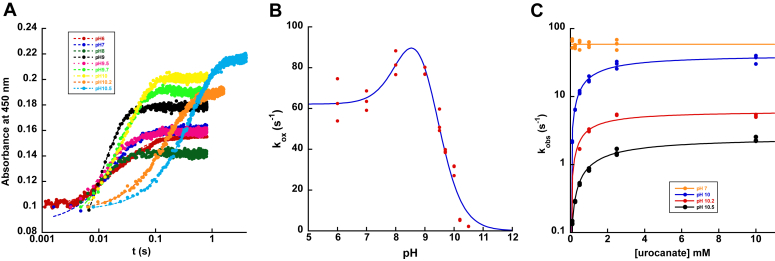
**pH dependence of the reaction between UrdA′-Fl**_**red**_**and urocanate.***A*, stopped-flow absorbance traces for the reaction between wildtype UrdA′-Fl_red_ and saturating concentrations of urocanate at different pH values. Note the logarithmic time base. Reaction traces have been adjusted such that they all start at the same absorbance to facilitate comparison. Note that the UrdA′ concentration was not identical at each pH (varied from 9 to 16 μM used), and the kinetic amplitudes are therefore not directly comparable. *B*, pH dependence of the rate constant for flavin oxidation. Fitting the data to Equation 4 yielded apparent p*K*a values of 8.2 ± 0.3 and 9.4 ± 0.1 and *k*_ox_ values of 62 ± 3 s^−1^ for the fully protonated state and 114 ± 13 s^−1^ for the singly ionized state. *C*, *k*_obs_ values plotted against the concentration of urocanate for pH values 7, 10, 10.2, and, 10.5. Note the logarithmic *y*-axis. The *k*_obs_ values for pHs 10, 10.2, and 10.5 showed a hyperbolic dependence on urocanate concentration, which was fitted to Equation 3. The *k*_ox_ and *K*_*d*_ values from fitting the data can be found in Table 1. UrdA, urocanate reductase.

To investigate the pH dependence of the oxidative half-reaction for UrdA′, UrdA′-Fl_red_ was oxidized by varying urocanate concentrations at different pH values from pH 6 to pH 10.5 under transient-state conditions (Fig. 8*A*). Values of pH exceeding 10.5 were not utilized because of the enzyme's susceptibility to unfolding, as evidenced by CD measurements (Fig. S2).

Most of the stopped-flow data (except for pH 6 and pH 7) fit to a single exponential function (Equation 1), attributed to the conversion of UrdA′-Fl_red_–urocanate to UrdA′-Fl_ox_–ImP, indicating that the step for product release is not observable at pH ≥8. The UrdA′-Fl_ox_–urocanate complex at pH 9 still has a greater absorbance at 450 nm than the UrdA′-Fl_ox_–ImP complex (Fig. S3), suggesting that the rate constant for product release may increase at high pH to the point that this step is no longer observable in the kinetic traces. The rate constant for flavin oxidation (*k*_ox_) was found to be maximal at pH 8 to 9 and decreased rapidly as the pH increased above this, approaching zero at pH 10.5 (Fig. 8*B*). Curiously, *k*_ox_ at pH 6 to 7 was slightly but reproducibly lower than it was at pH 8 to 9. Thus, the pH dependence of *k*_ox_ fit best to a model where two titratable groups affect *k*_ox_ for the reaction of UrdA′-Fl_red_ with urocanate, but the protonated form of the group with the lower apparent p*K*_a_ value (p*K*_a1_) reacts with a nonzero *k*_ox_ value (Equation 4) (17, 18). The fit yielded values of 8.2 ± 0.3 for p*K*a_1_ and 9.4 ± 0.1 for the higher p*K*_a_ value (p*K*_a2_) controlling flavin oxidation. We note that these are apparent p*K*a values since they are less than two pH units apart and therefore do not represent true p*K*a values.

A noteworthy observation is that at pH 10 and higher, the plots of *k*_obs_ against urocanate concentration display a hyperbolic dependence and require much higher urocanate concentrations to reach saturation (Fig. 8*C*). In contrast, *k*_obs_ was invariant with urocanate concentration at pH 9.5 and below, consistent with the stronger binding affinity observed at pH 7. Consequently, the *K*_*d*_ values obtained from fitting the *k*_obs_ plots increase as the pH increases (Table 1). This weaker binding affinity for urocanate at higher pH values may be attributed to Arg560, which forms a salt bridge with the carboxylate of urocanate, and this interaction would be disrupted when Arg560 gets deprotonated at higher pH values.

**Table 1:** Kinetic parameters for the reaction between wildtype UrdA′-Fl_red_ and urocanate at select pH values^a^

pH	7	10	10.2	10.5
*k*_ox_	59 ±6 s^−1^	30 ± 2 s^−1^	5.4 ± 0.2 s^−1^	2.2 ± 0.1 s^−1^
*K*_*d*_	ND	1130 ± 180 μM	900 ± 220 μM	1760 ± 190 μM

ND, not determined.

a At 4 °C.

### Mutation of active-site residues

**Figure 9:**
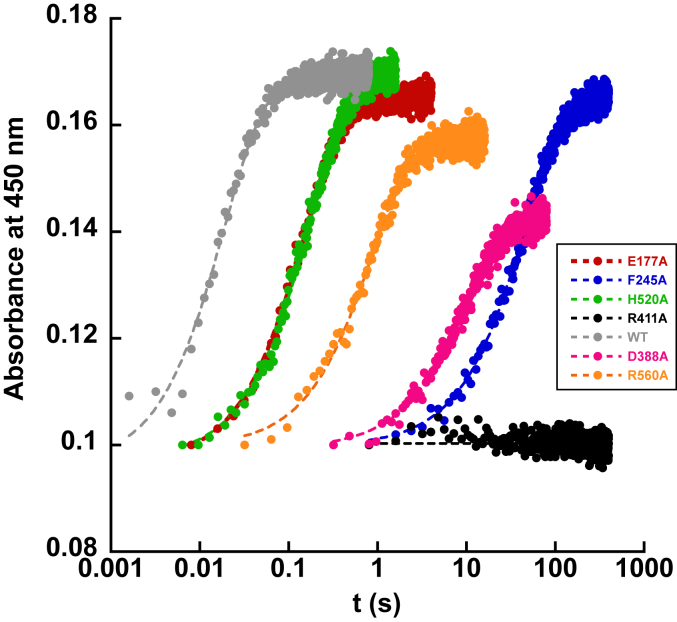
**Stopped-flow absorbance traces for the oxidative half-reaction of UrdA′ mutants with saturating concentrations of urocanate at pH 7**. Note the logarithmic time base. The *k*_ox_ and *K*_*d*_ values from fitting reaction traces can be found in Table 2. About 15 μM enzyme was used for each variant, and reaction traces have been adjusted such that they all start at the same absorbance to facilitate comparison. UrdA, urocanate reductase.

The X-ray structure of UrdA′ in complex with urocanate (Protein Data Bank ID: 6T87) shows a number of interactions between the substrate and residues in the active site (Fig. 2*B*). The carboxylate group of urocanate interacts extensively through a salt bridge to Arg560, a hydrogen bond to Arg411, and another hydrogen bond to His520. Arg411 is the proposed proton donor that delivers a proton to C2 of urocanate during catalysis and is situated 3.6 Å away from urocanate's C2 carbon. Urocanate’s imidazole ring nitrogen atoms can also establish hydrogen bonds with and depending on the protonation state of urocanate’s imidazole, salt bridges with Glu177 and Asp388. The residue Phe245 is close to the substrate (3.9 Å) and is part of an aromatic lid that closes over the active site upon substrate or product binding and has been proposed to help with substrate binding and product release and excluding solvent from the active site (10). To assess the function of these residues in close proximity to urocanate, we individually mutated them to alanine and analyzed the reaction of the reduced mutant enzymes with urocanate by anaerobic stopped-flow experiments. Stopped-flow traces at 450 nm for all the mutants at saturating urocanate concentrations can be found in Figure 9, and all could be fit to a single exponential function. Data for *k*_ox_ and *K*_*d*_ values, where measured, for the mutants can be found in Table 2.

**Table 2:** Kinetic parameters for the reaction between UrdA′-Fl_red_ mutants and urocanate^a^

Parameter	Wildtype	His520Ala	Glu177Ala	Arg560Ala	Asp388Ala	Phe245Ala
*k*_ox_	59 ± 6 s^−1^	5.9 ± 1.2 s^−1^	6.5 ± 0.2 s^−1^	1.1 ± 0.1 s^−1^	0.10 ± 0.01 s^−1^	0.022 ± 0.003 s^−1^
*K*_*d*_	ND	380 ± 80 μM	ND	3300 ± 900 μM	ND	83 ± 24 μM

ND, not determined.

a At pH 7 and 4 °C.

**Figure 10:**
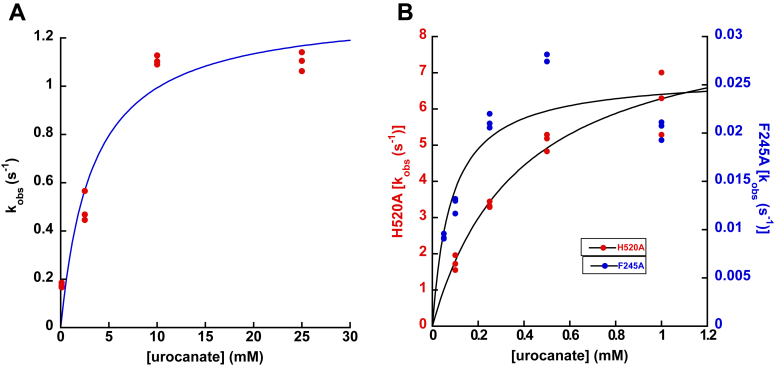
**Plots of *k***_**obs**_**for UrdA′ mutants with reduced affinity for urocanate.***A*, *k*_obs_ values plotted against the concentration of urocanate for the mutant Arg560Ala. The depicted graph exhibits a distinct hyperbolic relationship fitted to Equation 3, and this mutant necessitates considerably higher urocanate concentrations to achieve saturation of *k*_obs_ during the reaction. *B*, *k*_obs_ values plotted as a function of urocanate concentration for His520Ala and Phe245Ala also show a hyperbolic dependence, indicating higher *K*_*d*_ values for these mutants. UrdA, urocanate reductase.

Mixing urocanate with the Arg411Ala mutant containing FADH_2_ failed to oxidize the flavin at all even after waiting for several minutes, indicating that hydride transfer from FADH_2_ to urocanate is completely blocked by this mutation. This observation is consistent with the proposed critical role of Arg411 in serving as the general acid that protonates C2 of urocanate during catalysis and indicates that hydride transfer from FADH_2_ to C3 of urocanate does not readily occur without the presence of this proton donor. Notably, the charge-transfer absorbance seen in wildtype UrdA′ was not observed with the Arg411Ala variant when mixing enzyme containing reduced flavin with urocanate (Fig. S4*A*). His520 and Arg560 interact with the carboxylate of urocanate, and *k*_ox_ was 10-fold and 55-fold lower for the His520Ala and Arg560Ala mutants, respectively. Plots of *k*_obs_ against urocanate concentration for these mutants showed a clear hyperbolic dependence, and Arg560Ala required much higher urocanate concentrations to saturate *k*_obs_ for the reaction (Fig. 10, *A* and *B*), indicating that this mutant binds urocanate with a much weaker affinity than wildtype UrdA'. Fitting *k*_obs_ plots for these mutants to Equation 3 returned *K*_*d*_ values of 380 μM for the His520Ala mutant and 3300 μM for the Arg560Ala mutant. In agreement with this weaker binding affinity, the absorbance spectrum for the reaction with Arg560Ala at the end of the dead time lacks the charge-transfer signal at 550 nm observed with wildtype, and the His520Ala mutant shows a diminished amount of this charge-transfer signal (Fig. S4, *B* and *C*). Glu177 and Asp388 interact with the imidazole of urocanate. The *k*_ox_ value for the Glu177Ala mutant was ninefold lower than that of wildtype, and *k*_obs_ for this mutant was invariant with urocanate concentration, suggesting that this mutant still binds urocanate with comparable affinity to wildtype. Consistent with this, absorbance spectra at completion of the dead time for the reaction of urocanate with the Glu177Ala mutant show considerable charge-transfer absorbance (Fig. S4*D*). The Asp388Ala mutant had a *k*_ox_ value 600-fold lower than wildtype, indicating that reactivity with urocanate is severely compromised by this mutation. Notably, *k*_obs_ was again invariant with urocanate concentration, suggesting that there is not a major change in binding affinity for urocanate with the Asp388Ala mutation. The dramatic decline in *k*_ox_ with the Asp388Ala mutation may be due to suboptimal positioning of urocanate for catalysis in this mutant. Though the reaction with urocanate is severely compromised in the Asp388Ala variant, this variant still showed evidence for the charge-transfer absorbance at 550 nm at the start of the reaction (Fig. S4*E*). Phe245Ala UrdA′ had the lowest *k*_ox_ value (∼2700-fold lower than wildtype) and took around 250 s to for the reaction to complete. The plot of *k*_obs_ against urocanate concentration for Phe245Ala UrdA′ displayed a hyperbolic dependence; it also indicated that this mutant enzyme binds urocanate with moderately reduced affinity (83 μM) compared with wildtype (Fig. 10*B*), and no charge-transfer signal was present at the start of the reaction with this variant (Fig. S4*F*). As previously noted, Phe245 is close to urocanate (⁓3.9 Å) and is part of the aromatic lid in the active site of UrdA, which is not conserved in fumarate reductase.

## Discussion

UrdA provides bacteria with an alternative terminal electron acceptor for respiration in the form of urocanate when O_2_ is limited, and this enzyme is structurally and functionally homologous to Fcc3 fumarate reductase, which enables bacteria to use environmental fumarate as a respiratory electron acceptor (4, 10). UrdA present in gut bacteria produces ImP upon reducing urocanate, which is a marker associated with type 2 diabetes, linking this gut microbial enzyme with a human metabolic disorder (1); thus, gaining a deeper comprehension of the underexplored UrdA enzyme is relevant for human health. Our studies described here investigated aspects of the catalytic mechanism of urocanate reduction by UrdA′ and indicate several mechanistic parallels between it and fumarate reduction by the well-studied Fcc3. Notably, Fcc3 function has primarily been investigated under steady-state conditions using excess artificial electron donors, whereas we investigated the mechanism of UrdA by monitoring the absorbance spectrum of its flavin in half-reactions without the potential complications of turnover (19, 20, 21, 22, 23). We presume this was due to the presence of four heme cofactors in full-length Fcc3, whose absorbance spectra overlap with that of the flavin unlike with the isolated FAD domain of UrdA′ used here.

Our stopped-flow analysis of the reaction of UrdA′-Fl_red_ with urocanate at pH 7 revealed the presence of three spectrally distinguishable flavin intermediates along the way to forming UrdA′-Fl_ox_ and ImP. The first formed within the dead time of the instrument and had spectral features consistent with a charge-transfer complex involving reduced flavin. This likely corresponds to a charge-transfer complex between anionic flavin hydroquinone (FADH^–^) and urocanate and indicates that urocanate rapidly binds to UrdA-Fl_red_ with an apparent rate constant >1000 s^−1^. Flavin oxidation then occurs, concomitant with urocanate reduction, at a rate constant of 59 s^−1^ to form the second spectrally distinguishable intermediate. The spectrum of this second intermediate is consistent with it containing oxidized flavin, which then converts to a third species containing a slightly higher absorbance with a rate constant of 8.6 s^−^^1^. Based on the differences in spectra between UrdA′-Fl_ox_ in the presence of urocanate and ImP (Fig. 6), this second spectral intermediate likely corresponds to a complex between UrdA′-Fl_ox_–ImP, and the ImP dissociates from the oxidized enzyme at a rate constant of 8.6 s^−1^ to be rapidly replaced by urocanate. The physiological electron donor for UrdA is unknown, and therefore, the kinetics of FAD reduction have yet to be characterized in full-length UrdA, so it remains unclear if the rate of ImP release limits turnover in the full-length enzyme. We note that it is conceivable the charge-transfer signal observed in the first intermediate could represent a complex between UrdA-Fl_ox_ and an ImP anion if both binding and hydride transfer occurred rapidly in the instrument’s dead time, followed by subsequent rate-limiting proton transfer from Arg411 to the ImP anion in the observable event with a rate constant of 59 s^−1^. However, the fact that no charge-transfer signal is seen when urocanate is mixed with UrdA-Fl_red_ in the Arg411Ala variant argues against this mechanism since only proton transfer is blocked in this variant and hydride transfer should still be possible if hydride transfer and proton transfer occurred in a stepwise fashion. Our data are thus most consistent with the first spectral intermediate after the instrument’s dead time representing a charge-transfer complex between UrdA-Fl_red_ and urocanate as depicted in Figure 5.

The invariance of *k*_obs_ for flavin oxidation with urocanate concentration in Figure 4*D* indicates that UrdA′-Fl_red_ binds urocanate with a much higher affinity than the 160 μM *K*_*d*_ measured for urocanate binding to UrdA′-Fl_ox_. The greater affinity for urocanate when UrdA′ contains FADH^–^ requires that the reduction potential of FAD in UrdA′ be higher when in complex with urocanate than in the ligand-free form of the enzyme. The reduction potential for the FAD in ligand-free full-length UrdA has been measured as having a midpoint reduction potential (*E*_m_) of −279 mV at pH 7.5, whereas the *E*_m_ for urocanate–ImP has been estimated at being ∼0 mV (4); thus, there would still be sufficient reducing power for urocanate reduction with a moderate increase in the *E*_m_ of FAD upon binding urocanate. Notably, the −279 mV *E*_m_ of the FAD in ligand-free full-length UrdA is lower than the measured *E*_m_ of the covalently bound FMN (∼−225 to −251 mV) in the N-terminal domain, which was initially curious given that electrons are thought to flow from donors in the membrane to FMN, then to FAD, and finally to urocanate during catalysis with full-length UrdA. Our data indicate the *E*_m_ for FAD is higher when bound to urocanate, which should facilitate electron flow in the expected direction from the FMN to FAD. We cannot determine the increase in *E*_m_ of the FAD upon binding urocanate without knowing the true *K*_*d*_ value for urocanate binding to UrdA-Fl_red_; however, it is tempting to speculate that this increase in *E*_m_ with substrate binding may be a mechanism of suppressing electron flow to the FAD domain of UrdA when urocanate is absent while also promoting substrate binding when the flavin is reduced and product release when the flavin is oxidized.

The pH dependence of the apparent rate constant for flavin oxidation indicates that the protonation state of two ionizable groups impacts the rate constant for the oxidation of UrdA′-Fl_red_ by urocanate. Deprotonation of the group or groups with the net apparent p*K*a of 8.2 increases *k*_ox_ ∼1.8-fold. The modest change in *k*_ox_ associated with protonation–deprotonation of this group indicates that its protonation status is not critical for catalysis but may have a subtle effect on optimizing the reaction between UrdA′-Fl_red_ and urocanate. The p*K*a for the imidazole moiety of urocanate is 6.1 in solution (24), and the p*K*a for this group would be expected to be higher when bound to UrdA′ given the proximity of the side chains of Glu177 and Asp388 to the imidazole moiety of urocanate in the crystal structure of the complex. However, the protonation status of the imidazole moiety of urocanate would not be expected to be critical for urocanate reduction by UrdA′-Fl_red_, and the subtle difference in *k*_ox_ associated with apparent p*K*a_1_ may conceivably be reporting on the protonation–deprotonation of the imidazole moiety of urocanate when bound to UrdA’. The pH dependence of *k*_cat_ for fumarate reduction in *Shewanella frigidimarina* Fcc3 shows a p*K*a of 7.4 for an ionizable group where deprotonation decreases *k*_cat_ ∼4-fold, most likely attributable to His365, which interacts with one of the two carboxylates of fumarate in the structure of Fcc3 (23). Notably, Fcc3 is not inactivated by deprotonation of this group, as *k*_cat_ for the enzyme is still 210 s^−1^ at pH 9. UrdA has Tyr373 at the equivalent position to His365 in *S. frigidimarina* Fcc3; Tyr373 in UrdA′ does not form specific contacts with urocanate but is positioned ∼4 Å away from urocanate’s imidazole moiety, and it is therefore possible that the apparent p*K*a of 8.2 measured for UrdA′ is reporting on the protonation status of Tyr373. Deprotonation of the group in UrdA′ with an apparent p*K*a of 9.4 reduces *k*_ox_ to near zero, indicating that the protonated form of this group is essential for the enzyme to react with urocanate. This observation is consistent with the proposed role of Arg411 in serving as the general acid that protonates C2 of urocanate alongside hydride transfer from FADH^–^ to C3 of urocanate, and it is therefore reasonable to conclude that the apparent p*K*a of 9.4 is reporting on the protonation state of Arg411. Arginine is strictly conserved at this position for Fcc3s and UrdAs, and this arginine has been shown to be essential for fumarate reduction by Fcc3s as it serves as the general acid that protonates C2 of fumarate in these enzymes (23). Mutation of Arg411 to alanine in UrdA′ completely abolishes the conversion of urocanate to ImP (10), and our stopped-flow data indicate that this mutation also blocks hydride transfer from FADH^–^ to urocanate. The Arg411Ala variant still binds and copurifies with FAD, suggesting it is properly folded while being inactive toward urocanate, though we cannot rule out the possibility that this mutation disrupts the active site in some way. However, that hydride transfer is blocked in Arg411Ala UrdA′ suggests that hydride transfer from FADH^–^ to C3 and proton transfer from Arg411 to C2 of urocanate may be concerted.

Urocanate is naturally a planar molecule in solution because of the conjugated network of double bonds extending from the carboxylate through the imidazole. However, in the structure of UrdA′, urocanate in the active site is bound in a twisted conformation where the imidazole moiety is twisted out of the plane from the rest of the molecule (Figs. 2*B* and S5). A similar twisted conformation has been observed for fumarate (also naturally planar) bound in the active site of Fcc3 (Fig. 2*A*). This twisted conformation for fumarate in Fcc3 has been proposed to promote nucleophilic attack by the hydride of the flavin N5 by weakening the conjugated double bonds in fumarate, and reduction to nonplanar succinate also relieves the torsional strain imposed on fumarate (11, 13, 25). A similar mechanism is likely operational in UrdA given that urocanate is twisted in the active site, and this phenomenon may help explain the outsized effects on *k*_ox_ that occur upon mutating some of the residues that interact with urocanate. Asp388 and Arg560 in UrdA′ bracket the two ends of the substrate-binding site and form salt bridges with the imidazole and carboxylate moieties of urocanate, respectively. Asp388 appears to be the primary residue responsible for holding the imidazole moiety in a twisted conformation out of plane of the rest of urocanate; this may explain the ∼600-fold lower *k*_ox_ observed in Asp388Ala UrdA′ as removal of Asp388 from the active site would be expected to reduce the torsional strain on urocanate, impairing reduction of urocanate by the enzyme. Similarly, Arg560 holds the carboxylate end of urocanate in place to maintain the twist throughout urocanate and Arg560Ala UrdA′ has a 60-fold lower *k*_ox_ than wildtype, and the weakest binding affinity for urocanate of any of the variants tested. Phe245 forms part of the hydrophobic lid that closes over the active site upon urocanate binding to hold the substrate in place and exclude solvent from most of the active site, and mutation of Phe245 to Ala leads to a ⁓700-fold drop in *k*_ox_. Glu177 and His520 have less direct interactions with urocanate than the other residues to maintain the substrate in its twisted conformation, and the Glu177Ala and His520Ala UrdA′ variants each display only a ∼10-fold decline in *k*_ox_ compared with wildtype as a result.

**Figure 11:**
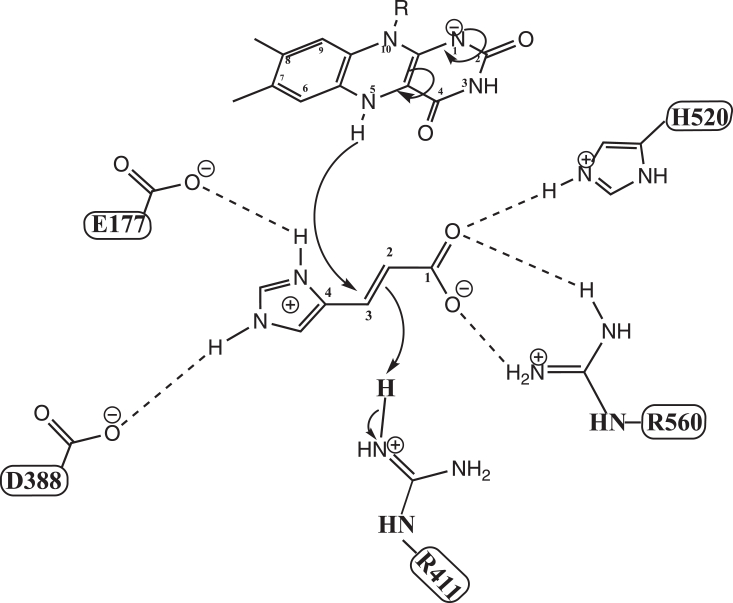
**Proposed mechanism of urocanate reduction by UrdA.** The substrate binds in a twisted nonplanar conformation through interactions with charged residues. This arrangement facilitates hydride transfer from flavin N5 to urocanate C3, and Arg411 serves as the active site acid, donating a proton to urocanate C2. UrdA, urocanate reductase.

We propose the mechanism depicted in Figure 11 for urocanate reduction by UrdA′-Fl_red_, which is consistent with most of our data. Urocanate binds in the enclosed active site in a twisted nonplanar conformation that is enforced by electrostatic interactions with active-site residues including Asp388 and Arg560. This positions C3 of urocanate in front of the flavin N5, and the twisted conformation weakens the conjugation between the double bonds in urocanate, facilitating hydride transfer from N5 of reduced flavin to the C3 of urocanate. Hydride transfer also eliminates the torsional strain imposed on urocanate by the binding site, and Arg411, which must be protonated for catalysis, is strategically located to provide a proton to C3 of the substrate, leading to the creation of ImP.

## Experimental procedures

### UrdA′ expression and purification

The UrdA′ gene from *S. oneidensis* MR-1, which includes the FAD-binding domain, and the mobile domain (amino acids 130–582) was purchased from Twist Biosciences and cloned into the NdeI and XhoI sites of pET29b to generate a construct with a C-terminal His tag. Subsequently, the vector was introduced into *E. coli* BL21 (DE3) cells and cultivated in expression media consisting of 12 g/l tryptone, 24 g/l yeast extract, 40 ml/l glycerol, 12.54 g/l K_2_HPO_4_, 2.13 g/l KH_2_PO_4_, and 50 mg/ml kanamycin at 37 °C with agitation until reaching an absorbance of 0.8 at 600 nm. The temperature was then reduced to 20 °C, and the cultures were induced with 500 μM IPTG and allowed to grow overnight. After harvesting the cultures, pellets were resuspended in a lysis buffer containing 300 mM NaCl, 50 mM NaPO_4_, 10% glycerol, and 20 mM imidazole, at pH 8. The cells were then lysed using sonication, with the addition of an EDTA-free protease inhibitor cocktail from Abcam and Benzonase nuclease from Sigma. The lysate underwent centrifugation, and the resulting supernatant was loaded onto a nickel affinity column that had previously been equilibrated with lysis buffer. A total of 100 ml of lysis buffer was used to wash the column (10 column volumes), and subsequently, UrdA′ was eluted using lysis buffer containing 250 mM imidazole. UrdA′ was concentrated and then subjected to additional purification by passing it through a HiLoad 16/600 Superdex 75 pg column at 4 °C. The purification process was carried out using a buffer consisting of 40 mM Hepes, 100 mM NaCl, and 10% glycerol, at pH 7.5. The resulting purified protein was then concentrated and flash frozen for storage at −80 °C. UrdA′ was exchanged into the appropriate buffer using an Econo-Pac 10DG desalting column (Bio-Rad) prior to performing an experiment.

### Site-directed mutagenesis

Single mutants of UrdA′ were prepared using site-directed mutagenesis (following standard protocols) to replace Arg540, His520, Arg411, Glu177, Asp388, and Phe245 with alanine in the original C-terminal 6xHis-tagged pET29b-based construct. The introduced mutations were verified by sequencing to ensure their accuracy, and the mutant enzymes were purified using the same protocol for wildtype. A representative SDS-PAGE gel is shown in Fig. S6 to demonstrate enzyme purity.

### Extinction coefficients

Extinction coefficients at 450 nm, pH 7, were determined for wildtype UrdA and all variants by denaturing the enzyme with 1% SDS and 95 °C treatment for 5 min to release free FAD. The extinction coefficient of FAD bound to the variants was then calculated from the predenaturation and postdenaturation spectra and using the known ε_450_ of 11,300 M^−1^ cm^−1^ for free FAD. The ε_450_ values of UrdA′ determined were 11,700 M^−1^ cm^−1^ for wildtype, 10,900 M^−1^ cm^−1^ for Glu177Ala, 12,100 M^−1^ cm^−1^ for Phe245Ala, 12,300 M^−1^ cm^−1^ for Asp388Ala, 12,000 M^−1^ cm^−1^ for Arg411Ala, 13,000 M^−1^ cm^−1^ for His520Ala, and 11,400 M^−1^ cm^−1^ for Arg560Ala.

### Flavin reduction

The flavin of UrdA′ was reduced with dithionite prior to performing any experiment on the oxidative half-reaction. A UrdA' solution with an absorbance of 0.3 to 0.4 at 450 nm (25–35 μM) was made anaerobic in a tonometer, where the removal of oxygen was achieved by cycling the tonometer between vacuum and anaerobic argon environments (26). Subsequently, the UrdA′ solution was subjected to anaerobic titration using a dithionite solution (1 mg/ml) in order to reduce the flavin present. Note that the stock concentration of dithionite is not truly known for this titration since some dithionite will react with O_2_ during aerobic preparation. The dithionite solution was made with the same buffer used for the enzyme and substrate and was placed in a gas-tight Hamilton syringe and added incrementally until the flavin in UrdA reached a fully reduced state. The absorption of UrdA's flavin was continuously monitored during the titration using a Cary 50 Bio UV–Visible Spectrophotometer and Cary WinUV software. The same procedure was followed to fully reduce the flavin in mutant variants as well.

### Transient kinetic assays

Stopped-flow experiments were completed at 4 °C using a TgK Scientific SF-61DX2 KinetAsyst stopped-flow instrument. All stopped-flow experiments were monitored using the instrument’s multiwavelength CCD detector. The standard buffer for experiments comparing mutant enzymes with wildtype was 50 mM Bis–Tris propane, 100 mM NaCl, 10% glycerol, at pH 7. 50 mM Bis–Tris propane was also used for pH values from 6 to 9 for the pH-dependent experiments. 50 mM CAPS was used in place of Bis–Tris propane for experiments at pH >9. Anaerobic reduced UrdA′ prepared in a tonometer as described previously was loaded onto the instrument, and the enzyme was mixed against various concentrations of urocanate in the same buffer as enzyme that had been sparged with argon to achieve anaerobiosis.

The data obtained from the stopped-flow experiments were analyzed using KaleidaGraph (Synergy software). The traces were fitted to either a single exponential function (Equation 1) or a two-exponential function (Equation 2). This fitting process allowed for the determination of observed rate constant (*k*_obs_) values for each urocanate concentration. In Equations 1 and 2, the kinetic amplitude for each phase is represented by ΔA, the apparent first-order rate constant is denoted as *k*_obs_, and A_∞_ refers to the absorbance at the completion of the reaction.

(1) $$Y=\Delta Ae^{-k_{οbs}t}+A\infty$$

(2) $$Y=\Delta A_{1}e^{-k_{obs1}t}+\Delta A_{2}e^{-k_{obs2}t}+A\infty$$

The plot of *k*_obs_ against [urocanate] for certain pH values and mutant enzymes showed a hyperbolic dependence and was fit to Equation 3 to determine *k*_ox_, the rate constant for flavin oxidation, and *K*_*d*_ for urocanate binding to UrdA′-Fl_red_.

(3) $$k_{obs}=\frac{k_{ox}\left[S\right]}{k_{d}+\left[s\right]}$$

The effect of pH on *k*_ox_ for wildtype UrdA′ could be described by a model for a doubly ionizing system (Equation 4) (17).

(4) $$X=\frac{{\left[H^{+}\right]}^{2}X_{H_{2}A}+\left[H^{+}\right]K_{a1}X_{HA^{–}}+K_{a1}K_{a2}X_{A^{2–}}}{K_{a1}K_{a2}+\left[H^{+}\right]K_{a1}+{\left[H^{+}\right]}^{2}}$$

In Equation 4, X represents the experimental observable (in this case, *k*_ox_), X_H2A_, X_HA–_, and X_A2–_ represent the *k*_ox_ values for the fully protonated, singly ionized, and doubly ionized species in the titration, respectively, and *K*_a1_ and *K*_a2_ represent the two *K*_a_ values for the titratable observable. Fitting the data in Figure 8*B* directly to Equation 4 resulted in erroneously negative values for X_A2–_. Accordingly, X_A2–_ was fixed at 0 for the fit shown in Figure 8*B*.

### ITC

The ITC analysis was conducted using a MicroCal ITC200 instrument in 50 mM Bis–Tris propane, 100 mM NaCl, 10% glycerol, pH 7 at 4 °C using a stirring speed of 750 rpm. The experiments involved titrating 1 mM of urocanate into a cell containing UrdA′-Fl_ox_ (wildtype) at a concentration of 50 μM. The titration was performed using 2 μl injections spaced 3 min apart. A control titration of urocanate into buffer was also performed to account for the heat of urocanate dilution, and the resulting “blank” heat signals were subtracted from the data for the experiment containing UrdA′-Fl_ox_. Because of the weak binding affinity relative to the UrdA′ concentration used; the binding stoichiometry could not be accurately determined when fitting the data to the one set of sites model using the Origin data analysis software provided with the instrument. Accordingly, the binding stoichiometry was fixed at 1:1 urocanate:UrdA when fitting to the one-site model to determine the *K*_*d*_ and enthalpy of binding reported in Figure 7.

### CD spectroscopy

CD measurements were collected at room temperature using a 1 mm path-length cuvette in a Jasco J-815 Spectropolarimeter. Solutions of wildtype UrdA′ with the same concentrations of 3.33 μM were prepared in 10 mM CAPS buffer at pH values 9.5, 10.5, 11, and 12. For pH 7, UrdA′ was in 10 mM phosphate buffer.

## Data availability

All data are contained within the article.

## Supporting information

This article contains supporting information.

## Conflict of interest

The authors declare that they have no conflicts of interest with the contents of this article.

## References

[1] Koh A., Molinaro A., Ståhlman M., Khan M.T., Schmidt C., Mannerås-Holm L. (2018). Microbially produced imidazole propionate impairs insulin signaling through mTORC1. Cell.

[2] Wu B., Tan L., Wang W., Feng X., Yan D. (2022). Imidazole propionate is increased in diabetes and associated with stool consistency. Diabetes Metab. Syndr. Obes..

[3] Saad M.J.A., Santos A., Prada P.O. (2016). Linking gut microbiota and inflammation to obesity and insulin resistance. Physiology.

[4] Bogachev A.V., Bertsova Y.V., Bloch D.A., Verkhovsky M.I. (2012). Urocanate reductase: identification of a novel anaerobic respiratory pathway in Shewanella oneidensis MR-1. Mol. Microbiol..

[5] Molinaro A., Bel Lassen P., Henricsson M., Wu H., Adriouch S., Belda E. (2020). Imidazole propionate is increased in diabetes and associated with dietary patterns and altered microbial ecology. Nat. Commun..

[6] Karlsson F.H., Tremaroli V., Nookaew I., Bergström G., Behre C.J., Fagerberg B. (2013). Gut metagenome in European women with normal, impaired and diabetic glucose control. Nature.

[7] Qin J., Li Y., Cai Z., Li S., Zhu J., Zhang F. (2012). A metagenome-wide association study of gut microbiota in type 2 diabetes. Nature.

[8] Larsen N., Vogensen F.K., Van Den Berg F.W.J., Nielsen D.S., Andreasen A.S., Pedersen B.K. (2010). Gut microbiota in human adults with type 2 diabetes differs from non-diabetic adults. PLoS One.

[9] Light S.H., Méheust R., Ferrell J.L., Cho J., Deng D., Agostoni M. (2019). Extracellular electron transfer powers flavinylated extracellular reductases in Gram-positive bacteria. Proc. Natl. Acad. Sci. U. S. A..

[10] Venskutonytė R., Koh A., Stenström O., Khan M.T., Lundqvist A., Akke M. (2021). Structural characterization of the microbial enzyme urocanate reductase mediating imidazole propionate production. Nat. Commun..

[11] Leys D., Tsapin A.S., Nealson K.H., Meyer T.E., Cusanovich M.A., Van Beeumen J.J. (1999). Structure and mechanism of the flavocytochrome c fumarate reductase of Shewanella putrefaciens MR-1. Nat. Struct. Biol..

[12] Pankhurst K.L., Mowat C.G., Rothery E.L., Hudson J.M., Jones A.K., Miles C.S. (2006). A proton delivery pathway in the soluble fumarate reductase from Shewanella frigidimarina. J. Biol. Chem..

[13] Taylor P., Pealing S.L., Reid G.A., Chapman S.K., Walkinshaw M.D. (1999). Structural and mechanistic mapping of a unique fumarate reductase. Nat. Struct. Biol..

[14] Massey V. (2000). The chemical and biological versatility of riboflavin. Biochem. Soc. Trans..

[15] Palfey B.A., Björnberg O., Jensen K.F. (2001). Insight into the chemistry of flavin reduction and oxidation in Escherichia coli dihydroorotate dehydrogenase obtained by rapid reaction studies. Biochemistry.

[16] Fagan R.L., Jensen K.F., Björnberg O., Palfey B.A. (2007). Mechanism of flavin reduction in the class 1A dihydroorotate dehydrogenase from Lactococcus lactis. Biochemistry.

[17] Fersht A. (1999). Structure and Mechanism in Protein Science.

[18] Beaupre B.A., Forouzesh D.C., Butrin A., Liu D., Moran G.R. (2021). Perturbing the movement of hydrogens to delineate and assign events in the reductive activation and turnover of porcine dihydropyrimidine dehydrogenase. Biochemistry.

[19] Mowat C.G., Moysey R., Miles C.S., Leys D., Doherty M.K., Taylor P. (2001). Kinetic and crystallographic analysis of the key active site acid/base arginine in a soluble fumarate reductase. Biochemistry.

[20] Mowat C.G., Pankhurst K.L., Miles C.S., Leys D., Walkinshaw M.D., Reid G.A. (2002). Engineering water to act as an active site acid catalyst in a soluble fumarate reductase. Biochemistry.

[21] Rothery E.L., Mowat C.G., Miles C.S., Walkinshaw M.D., Reid G.A., Chapman S.K. (2003). Histidine 61: an important heme ligand in the soluble fumarate reductase from Shewanella frigidimarina. Biochemistry.

[22] Turner K.L., Doherty M.K., Heering H.A., Armstrong F.A., Reid G.A., Chapman S.K. (1999). Redox properties of flavocytochrome c 3 from Shewanella frigidimarina NCIMB400. Biochemistry.

[23] Doherty M.K., Pealing S.L., Miles C.S., Moysey R., Taylor P., Walkinshaw M.D. (2000). Identification of the active site acid/base catalyst in a bacterial fumarate reductase: a kinetic and crystallographic study. Biochemistry.

[24] Roberts J.D., Yu C., Flanagan C., Birdseye T.R. (1982). A nitrogen-15 nuclear magnetic resonance study of the acid-base and tautomeric equilibria of 4-substituted imidazoles and its relevance to the catalytic mechanism of α-lytic protease. J. Am. Chem. Soc..

[25] Reid G.A., Miles C.S., Moysey R.K., Pankhurst K.L., Chapman S.K. (2000). Catalysis in fumarate reductase. Biochim. Biophys. Acta.

[26] Moran G.R. (2019). Anaerobic methods for the transient-state study of flavoproteins: the use of specialized glassware to define the concentration of dioxygen. Methods Enzymol..

