# Supplementary material for: Rapid reaction studies on the chemistry of flavin oxidation in urocanate reductase
Source: J Biol Chem. 2024 Jan 26;300(3):105689. doi: 10.1016/j.jbc.2024.105689 (PMC10882135; doi:10.1016/j.jbc.2024.105689)
Supplement: Supporting information [file mmc1.pdf]

Supporting Information

Rapid reaction studies on the chemistry of flavin oxidation in urocanate reductase

Niusha Delavari, Zhiyao Zhang, and Frederick Stull*

Department of Chemistry, 1903 W Michigan Ave, Western Michigan University, Kalamazoo, MI 49008

*Corresponding author; email: frederick.stull@wmich.edu

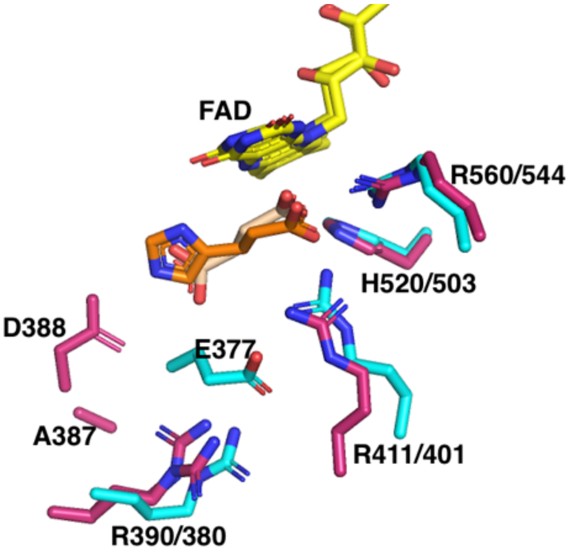

Figure S1. An overlay of the active sites of urocanate reductase in complex with urocanate ([1DB] ID: 6T87) and fumarate reductase in complex with fumarate ([PDB] ID: 1D4E, FAD (yellow), urocanate (orange), fumarate (wheat), urocanate reductase's active sire residues (magenta), fumarate reductase's active site residues (blue). In fumarate reductase R380, E377, and R401 form a proton delivery pathway.

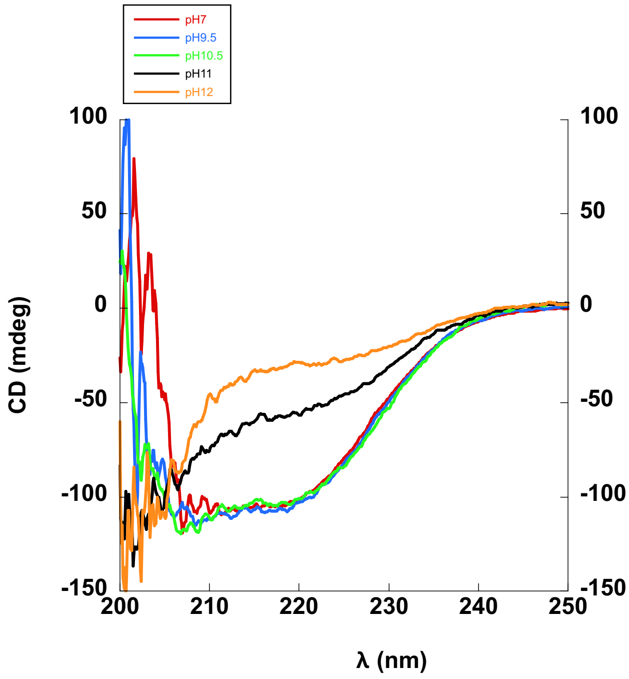

Figure S2. The circular dichroism (CD) plot at 25℃ with solutions of wild-type UrdA´ at different pH values.

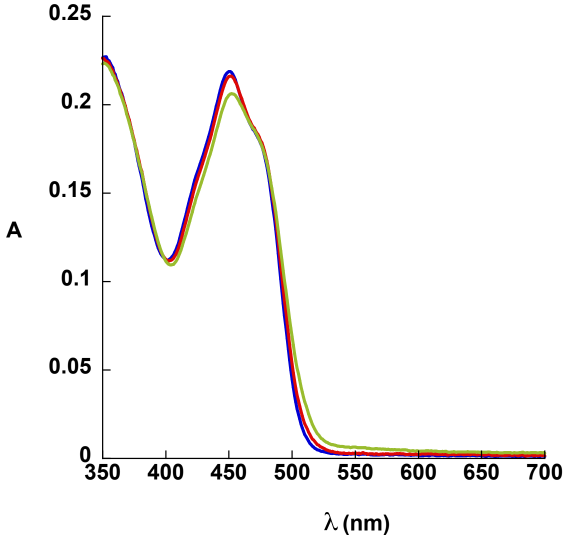

Figure S3. Absorbance spectra of UrdA'-Fl$_{ox}$ complexes at pH 9. Absorbance spectra for for UrdA´-Fl$_{ox}$ alone (blue), the UrdA′-Fl$_{ox}$-urocanate complex (red), the UrdA′-Fl$_{ox}$-imidazole propionate complex (green). The UrdA' concentration was 20 μM in all samples and 200 μM ligand was added for the spectra of each complex.

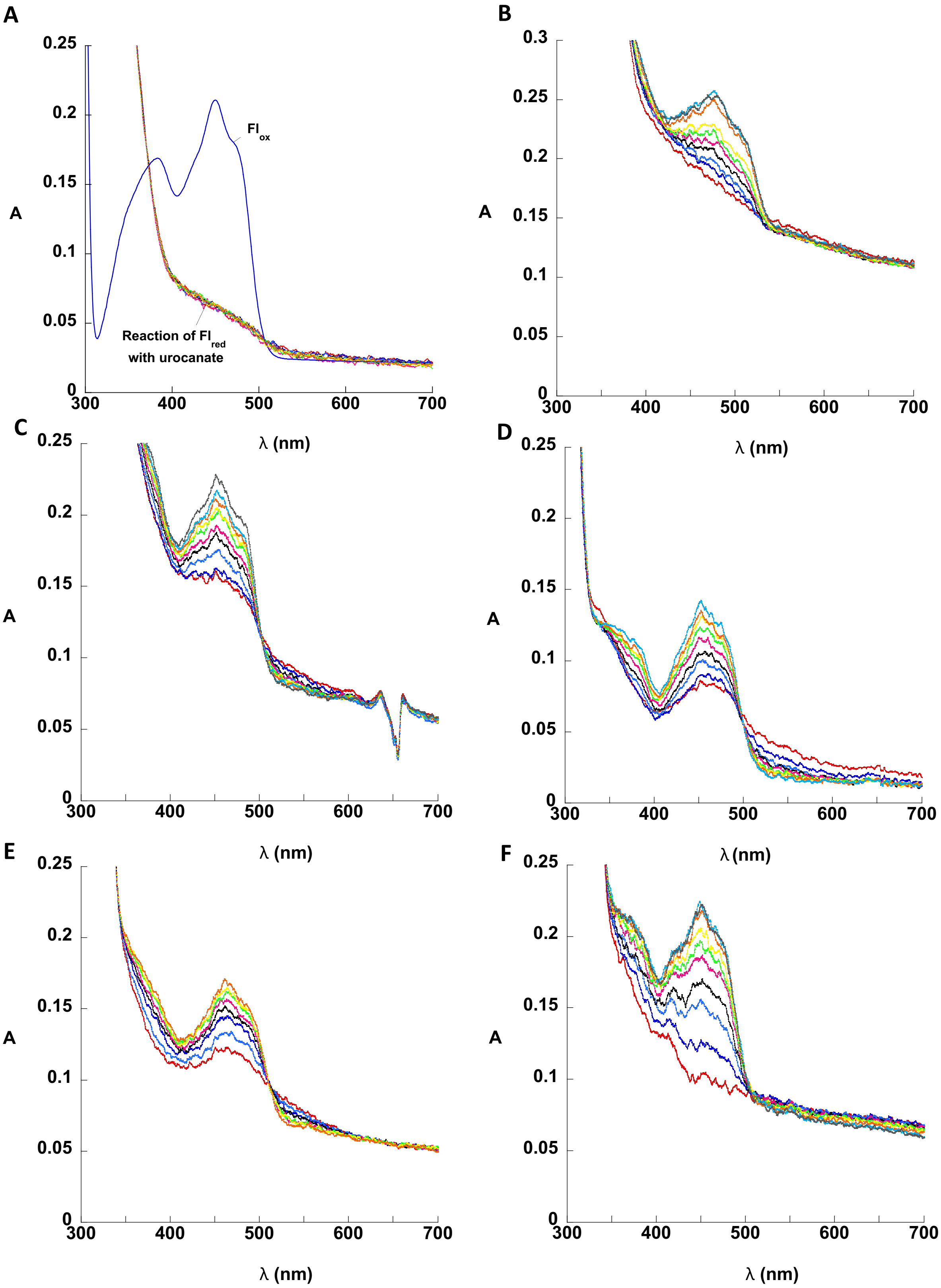
A
B
C
D
E
F
A
λ (nm)
Fl ox
Reaction of Fl red
with urocanate
0
0.05
0.1
0.15
0.2
0.25
0.3
300
400
500
600
700

Figure S4. Time-dependent absorbance spectra for the reaction between UrdA′-Fl_{red} mutants and saturating concentrations of urocanate. Plots of spectra illustrate the time-dependent re-oxidation of UrdA's reduced FAD by saturating concentrations of urocanate for different mutant types. Approximately 15 µM enzyme was used for each variant. (**A**) Mutant Arg411Ala (ligand free λ_{max} 450 nm). The absorbance spectrum of this variant containing oxidized FAD is shown for reference; (**B**) Mutant Arg560Ala (ligand free λ_{max} 454 nm); (**C**) Mutant His520Ala (ligand free λ_{max} 452 nm); (**D**) Mutant Glu177Ala (ligand free λ_{max} 457 nm); (**E**) Mutant Asp388Ala (ligand free λ_{max} 445 nm); (**F**) Mutant Phe245Ala (ligand free λ_{max} 453 nm). Regarding H520A, there exists an instrumentation artifact approximately at the wavelength of 650 nm.

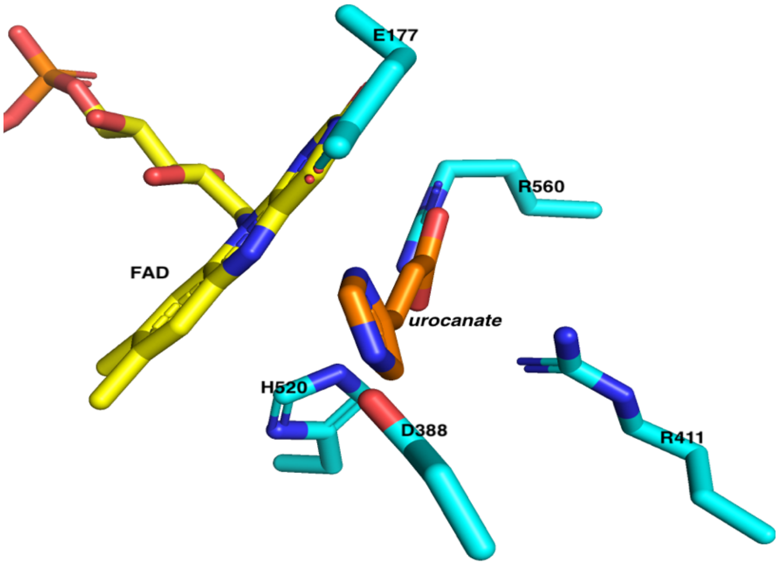

Figure S5. Twisted structure of urocanate in the active site of urocanate reductase (UrdA) [PDB 6T87]. In the active site, urocanate is bound in a twisted conformation, with the imidazole moiety twisted out of the molecule's plane.

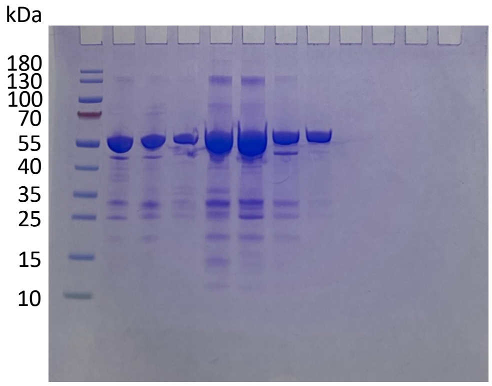

Figure S6. Representative SDS-PAGE gel to show purity of UrdA variants. Lane 1, PageRuler protein ladder. Lane 2, Arg560Ala UrdA’. Lane 3, Asp388Ala UrdA’. Lane 4, His520Ala UrdA’. Lane 5, Phe245Ala UrdA’. Lane 6, Glu177Ala UrdA’. Lane 7, Arg411Ala UrdA’. Lane 8, wild type UrdA’.
